# PSINDB: the postsynaptic protein–protein interaction database

**DOI:** 10.1093/database/baac007

**Published:** 2022-03-02

**Authors:** Zsofia E Kalman, Dániel Dudola, Bálint Mészáros, Zoltán Gáspári, Laszlo Dobson

**Affiliations:** Faculty of Information Technology and Bionics, Pázmány Péter Catholic University, Práter u. 50/A, Budapest 1083, Hungary; Faculty of Information Technology and Bionics, Pázmány Péter Catholic University, Práter u. 50/A, Budapest 1083, Hungary; Structural and Computational Biology Unit, European Molecular Biology Laboratory, Meyerhofstraße 1, Heidelberg 69117, Germany; Faculty of Information Technology and Bionics, Pázmány Péter Catholic University, Práter u. 50/A, Budapest 1083, Hungary; Structural and Computational Biology Unit, European Molecular Biology Laboratory, Meyerhofstraße 1, Heidelberg 69117, Germany; Institute of Enzymology, Research Centre for Natural Sciences, Magyar Tudósok Körútja 2, Budapest 1117, Hungary

## Abstract

The postsynaptic region is the receiving part of the synapse comprising thousands of proteins forming an elaborate and dynamically changing network indispensable for the molecular mechanisms behind fundamental phenomena such as learning and memory. Despite the growing amount of information about individual protein–protein interactions (PPIs) in this network, these data are mostly scattered in the literature or stored in generic databases that are not designed to display aspects that are fundamental to the understanding of postsynaptic functions. To overcome these limitations, we collected postsynaptic PPIs complemented by a high amount of detailed structural and biological information and launched a freely available resource, the Postsynaptic Interaction Database (PSINDB), to make these data and annotations accessible. PSINDB includes tens of thousands of binding regions together with structural features, mediating and regulating the formation of PPIs, annotated with detailed experimental information about each interaction. PSINDB is expected to be useful for various aspects of molecular neurobiology research, from experimental design to network and systems biology-based modeling and analysis of changes in the protein network upon various stimuli.

Database URL

https://psindb.itk.ppke.hu/

## Introduction

Synapses are communication points between neurons and thus are responsible for transducing information. Chemical synapses can be broadly split into presynaptic sites releasing neurotransmitters and the postsynaptic region, often referred to as postsynapse (PS), receiving and processing these incoming chemical signals. Cellular- and organism-level events, such as neuronal development, memory and long-term potentiation, strengthen synaptic connections (1). Excitatory PSs contain a special morphological unit called postsynaptic density (PSD), which localizes directly under the membrane of the receiving cell (2). The PSD is composed of thousands of interacting proteins forming an intricate network. While there are hallmark PPIs observed in all PSDs, the exact distribution and organization of components vary between neuron types and developmental stages (3). The PSD can also be dynamically restructured during the diurnal cycle (4) and by long-term potentiation (5). Mutations in postsynaptic proteins are responsible for a wide range of neurological and psychiatric diseases (6), many of them likely to be heritable as suggested by a growing number of evidence, such as in the case of autism spectrum disorder (7, 8).

Protein–protein interactions (PPIs) play an essential role both in the maintenance of synaptic plasticity and in disease emergence. Postsynaptic proteins exhibit a high degree of multivalency presenting multiple binding sites, and this multivalency aids the formation of an elaborate and dynamic network provided by distinct structural and functional elements (9). Intrinsically disordered regions were shown to be critical components for network assembly in the PS (10), often by containing short linear motifs mediating transient interactions (11) and post-translational modification sites regulating interactions as switches (12), among others.

Although there is a huge amount of information available on PPIs formed in the PS, they are either scattered in the literature or are collected in databases that were not designed to store complex information that aids the better understanding of synapse formation and function at the molecular level. Here, we present the Postsynaptic Interaction Database (PSINDB, https://psindb.itk.ppke.hu/) aiming to provide a more complete and complex picture of the postsynaptic protein network. PSINDB is a comprehensive database focused on postsynaptic proteins, containing a high number of collected, manually curated or derived interactions with precisely defined binding regions, enriched by various structural and functional features of the constituent proteins. We believe that PSINDB will facilitate model building and experimental design addressing the organization of the PS and thus will ultimately help scientists to get closer to the understanding of the complex nature of synaptic function at the molecular level.

## Results

### Statistics of the PSINDB database

**Figure 1.**
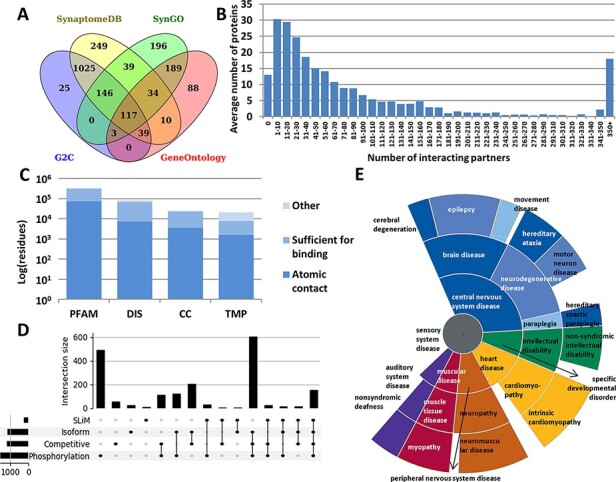
Summary statistics of PSINDB database: (A) Number of postsynaptic proteins from each source database and their overlaps. (B) Distribution of interacting partners of postsynaptic proteins. (C) Ratio of residues in different structural binding regions, colored by the level of information available about the binding residue (PFAM: belonging to protein families identified by Pfam; DIS: Disordered; CC: Coiled-coil; TMP: Transmembrane Protein). (D) Distribution of regulatory mechanisms in PS proteins (SLiM: Short Linear Motif). (E) Distribution of diseases associated with PS proteins. The more general the term, the closer it is to the center.

Every annotation in PSINDB is mapped to proteins in the human reference proteome. As the involvement of a specific protein in the PS is dependent on the cell type and developmental stage, there is no single comprehensive resource of PS proteins that would cover all aspects. Therefore, we integrated the PS protein definitions of four well-established databases, GeneOntology (13), SynGO (14), SynaptomeDB (15) and Gene2Cognition (16), to define the 2160 postsynaptic proteins (Figure 1A, see the ‘Methods’ section). 5.4% of these were included in all source databases, and 25.9% had annotation in only one of them.

We collected interactions from IntAct (17) and BioGRID (18), the Protein Data Bank (PDB) (19) and high scoring computational data from the STRING database (20) (see the ‘Methods’ section). We also annotated nearly 2000 postsynaptic interactions by manual curation based on PS literature. The majority of all PSINDB proteins (1177 proteins, 54.5%) have 1–50 interacting partners, 475 proteins (22%) bind to >100 unique partners, and for only 13 proteins (<1%) we could not find any interaction partner (Figure 1B).

In addition to annotating binary interactions between proteins, we also encode the exact binding regions where possible. To unify the different levels of evidence, we used the following terms from the HUPO-PSI Molecular Interaction ontology to classify binding regions: binding-associated regions, sufficient for binding and necessary for binding (see the ‘Methods’ section). We also included an additional level, namely atomic contacts, that refers to data derived from PDB interactions in atomic resolution. PSINDB contains binding region information at different levels: (i) for each experiment separately, (ii) for each partner (data from different experiments for the same partner are merged) and (iii) for each protein (binding regions to different partners are merged, displaying all interacting regions for the protein at once). Each entry in PSINDB also contains structural information about the protein (Figure 1C). Nearly all postsynaptic proteins exhibit high modularity (9), where the ordered and disordered modules mediate different interactions. Coiled-coils are also essential players in the PS: they often provide spacer and linker functions, aiding the formation of supramolecular assemblies by connecting distant compartments and proteins. Membrane proteins have critical importance, transmitting signals across the membrane. Proteins in these classes have strikingly different amounts of information available on interactions: while 20% of residues in structural domains are involved in interactions, for transmembrane proteins no detailed information is available.

To describe regulatory processes for the annotated interactions, PSINDB contains information on posttranslational modifications and alternative splicing (21). These mechanisms are prevalent and are often intertwined in PS proteins: >50% of the postsynaptic proteome contains at least one phosphorylation site and around one-third uses alternative splicing, competitive binding and phosphorylation, and nearly 10% of PS proteins use all four regulatory mechanisms (Figure 1D).

**Figure 2.**
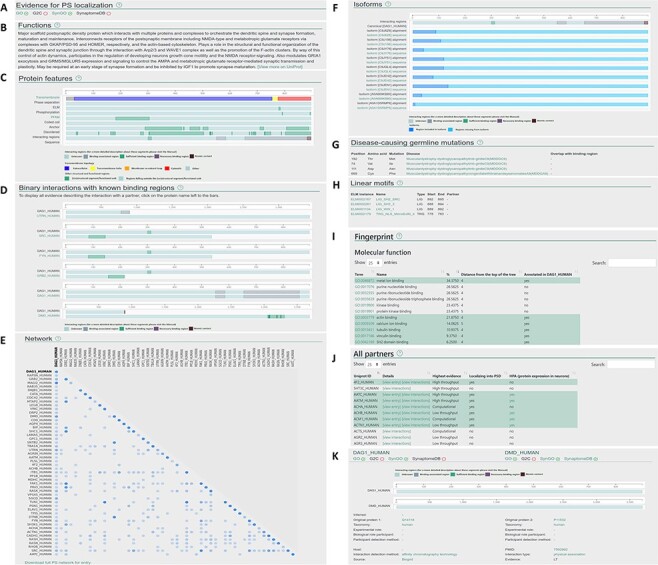
Layout of the individual entry pages (A–J) and the interaction pages (K) of PSINDB: Protein page: (A) Evidence for PS localization, (B) function, (C) protein features, (D) binary partners with known interacting regions, (E) network, (F) isoforms, (G) disease-causing germline mutations, (H) short linear motifs, (I) fingerprint, (J) all partners. Interaction page: (K) details of the experiment.

PSINDB also includes disease-associated germline mutations (22): many illnesses are related to nervous system diseases, and most of them belong to the central nervous system disease class. Interestingly, several mutations of PS proteins have been linked to heart and muscular diseases (Figure 1E), and PSINDB can enable the systematic inference of these disease connections and their mechanistic interpretations.

### Layout of the PSINDB database

PSINDB has an interactive graphical user interface for the visualization of interaction data. Data can be accessed either by browsing through the entries or via one of the two different types of searches.

The protein search will result in a list of proteins, while the interaction search will result in a list of interacting partners. In both cases, the gene name, Entrez and HUGO gene ID, UniProt ID and various aliases can be used as a query.

Clicking results from the protein search opens the individual protein entry pages, which includes a graphical overview of the corresponding protein along with 10 sections summarizing structural, interaction and network data (Figure 2A–J). The sections are as follows: (A) evidence for postsynaptic (PS) localization (with link to the source database) (B) function (short description mirrored from UniProt); (C) protein features (structural and interaction information, featuring interacting regions incorporating binding regions of all partners); (D) binary interactions with known binding regions (interacting regions with each partner one by one); (E) network (a matrix-like representation of partners, where the depth of the color encodes the strength of experimental evidence for the interaction); (F) isoforms (alignment of isoforms highlighting differences between them making it easily comparable with the location of binding regions); (G) disease-causing germline mutations (mutations and diseases associated with the protein, together with the partners where binding sites overlap with the mutated residue); (H) linear motifs (together with the partners where binding sites overlap with the mutated residue); (I) fingerprint (percentages of GeneOntology molecular function, GeneOntology biological process and Disease Ontology terms shared within the network of binary interaction partners of the protein); (J) all partners (including those not having postsynaptic annotations, yet the interactions indicate the partner may have the same localization).

The interaction page can be accessed from the protein page by clicking on the partner or from the results of the interaction search page. The interaction page contains the definition and details about the experiment (Figure 2K), including the type of interaction, interaction detection method, host organism, experimental and biological role of participants and more. Binding regions for each experiment are also displayed where available.

In addition to basic search options, we also offer several predefined subsets of postsynaptic proteins/interactions that may be interesting to a broad range of users. These set are defined based on higher-order assemblies: scaffold proteins, cytoskeletal proteins and hub proteins; based on structural features: transmembrane proteins, phase separation proteins and coiled-coil containing proteins; and based on evidence: literature and structural categories.

### Download options

Every interaction stored in PSINDB can be downloaded in a structured format. PSINDB uses the MITAB 2.5 format, which is standard in representing molecular interactions (23). We also added several other download options: the set of postsynaptic proteins (with their source), the postsynaptic network (as we defined it) and the binding regions between all partners are available for download. In addition, the network of individual proteins can be also accessed on every page.

## Discussion

### Example applications of PSINDB

The following examples highlight how PSINDB can serve as a central resource for PS-related protein research, far surpassing previous similar repositories in terms of both data volume and granularity. This enables both the in-depth study of individual proteins and protein sub-networks. In addition, PSINDB can also contribute to generating new, testable hypotheses concerning the interactions and functional roles of PS proteins.

**Figure 3.**
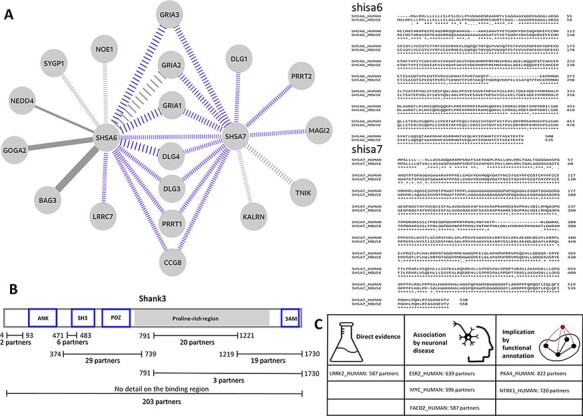
(A) Left: the interaction sub-network of SHSA6 and SHSA7. Edge weights encode the confidence of the interaction, blue edges show interactions manually curated into PSINDB and dashed lines represent interactions derived from studies on non-human homologues. Right: the alignment between human (top sequences) and mouse (bottom sequences) SHSA proteins. (B) Domain representation of SHANK3, together with the number of partners binding to the different regions of the protein as displayed in the PSINDB database. (C) Proteins not described as part of the PS in any of the source databases. Despite the lack of annotation, they interact with extremely high amounts of PS proteins, and several evidence hint they might localize to the PS or they might have an important role in the PS.

PSINDB can highlight local PS interaction networks in detail. Both shisa-6 and shisa-7 (SHSA6_HUMAN and SHSA7_HUMAN) are single pass transmembrane receptors located in the synapse membrane and are known to be involved in regulating transmission in CA3-CA1 synapses, possibly via regulating AMPA-type glutamate receptors. The existing interactions mapped for shisa-6 and shisa-7 were low in numbers, limiting the assessment of the full functional repertoire of these receptors based on easily accessible interaction data. Our manual curation efforts significantly extended the available interaction data for both receptors, adding 8 and 11 interactions for shisa-6 and shisa-7 on top of the already known 6 and 2 interactions. This means a total of 3.3-fold increase in the number of interactions for the two proteins. Figure 3A shows the current interaction network of these two receptors with newly annotated interactions in color. PSINDB allows for the assessment of interactions along different criteria, such as the reliability of the data encoded in the weight of the edges. This example also highlights how mapping interactions through close homologues can enrich interaction networks. Several high confidence interactions in this sub-network were derived from mouse studies, which can be reliably mapped to their human counterparts as the mouse and human SHSA proteins share a very high degree of sequence identity (Figure 3A).

Several PS-related proteins are extensively annotated in PSINDB via both integrating source data and adding manual curation. Members of the shank (SH3 and ankyrin repeat containing) protein family are among the most important postsynaptic scaffold proteins (24). These multidomain proteins can establish connections that link the layer of the membrane receptors to the cytoskeleton. Shank proteins are also capable of polymerization via their SAM domain (25) and can also form a network with Homer proteins (26). A number of shank mutations have been associated with diverse neurological disorders such as autism spectrum disorder (27), and these are often referred to as ‘Shankopathies’ (28). We have chosen shank3 (SHAN3_HUMAN) as an example of the family, for which 280 partners are available in PSINDB, with 77 of these having site-specific binding information. Two of the partners with known binding sites can bind to multiple regions on shank3, identified in the same high-throughput study (29): abi1 (ABI1_HUMAN) can bind both to the regions 374–739 and 791–1221, whereas gapdh (G3P_HUMAN) can interact with the segments 374–739 and 1219–1730. Hence, in Figure 3, the sum of partners with detailed annotation is 97, two more than the actual number of proteins for which binding region information is available. A recent study suggests alterations in the conformational landscape upon autism-associated mutations (30), and the distinct three-dimensional arrangements of shank3 are expected to expose the individual binding regions differently. The detailed region-specific annotations available in PSINDB (Figure 3B) make it possible to take the availability of interaction sites as well as the mutually exclusive nature of interactions at the same sites into account when creating models of postsynaptic protein complexes, e.g. similar to those described in Miski *et al.* (31).

Although most sections of the PSINDB database focus on interactions between PS proteins, we also display other partners without known PS localization in any of the source databases. PSINDB lists these proteins without a ‘PS’ tag; however, the lack of annotation is not proof for the protein not residing in the PS. Such proteins that do interact with a high number of PS-resident partners are likely candidates to be localized in the PS themselves. We assessed several of these proteins for possible indications of being PS localized, looking for annotations outside of the four source databases for localization (see the ‘Results’ section) (Figure 3C). Sometimes direct evidence can be found in the literature: for example, lrrk2 (LRRK2_HUMAN) has 587 postsynaptic partners, yet it was not included in any of the four source databases as a postsynaptic protein. According to Lee *et al.*, lrrk2 interacts with eif4ebp1 (4EBP1_HUMAN) at the PS in the neuromuscular junction (32). In several cases, no such direct evidence can be found; however, there are hints implicating postsynaptic roles: plekha4 (PKHA4_HUMAN) positively regulates the Wnt signalling pathway (33), which in turn regulates neurotransmitter receptors in the PS (34). Moreover, Wnt proteins promote postsynaptic recruitment, as shown in PSD-95 proteins (35) and GABAA receptors (36). Ntrk1 (NTRK1_HUMAN) also lacks postsynaptic evidence, yet it has over 700 PS partners. Ntrk1 is a nerve growth factor, and it was shown to play an important role in neuronal development and differentiation (37). In other cases, proteins with a high number of PS partners play a role in neuronal diseases: for example, esr2 (ESR2_HUMAN) (38) and myc (MYC_HUMAN) (39) are both associated with Alzheimerdisease, implicating synaptic roles. Notably, as these proteins are not part of our current postsynaptic definition, they cannot be found on PSINDB homepage; however, such lists can be easily generated using the files available in the Download section. Neuronal protein expression data are also available from the Human Protein Atlas (40), which can be used as an additional filtering layer when constructing such data sets. While none of this information alone is sufficient to declare these proteins to be parts of the PS, these proteins can be considered as candidates for further investigation.

### Comparison of PSINDB with other resources

PSINDB is a unique PPI database that provides various functionalities that are not readily available in other resources. The data are already narrowed down or can be filtered to be specific, e.g. PS localisation and neural expression is presented for each interacting partner of PS proteins. Since there is no complete agreement on the composition of the PS, four independent sources are used. Currently, only the database by Sorokrina *et al.* (41) offers similar filtering; however, their resource is limited to the synaptic annotations of SynGO and there is no publicly available web server to access their work.

PSINDB also provides a rich annotation for proteins to help users in better understanding the nature of interactions: (i) binding regions driving the interactions, together with (un)structural segments mediating the binding, (ii) functional processes playing a role in regulating the binding (linear motifs, PTMs, splicing) and (iii) functions and associated diseases of proteins and highlighting those that have importance considering the interaction network. Although some of these features are available in other databases (for example, IntAct also contains binding regions, STRING has a similar GO enrichment annotation displaying terms co-occurring in multiple partners of the same network and the resource by Sorokrina *et al.* also links diseases to proteins), all this information is not collected together in any other database. By integrating all this information into PSINDB, users do not have to use multiple prediction tools and databases when analyzing their protein/interaction of interest.

PSINDB focuses on the human postsynaptic interactome and therefore interactions from mouse and rat studies are all mapped to the human orthologs of the protein, providing a unique and useful tool for interactome analysis that is not limited to different species.

### Future directions

We plan to keep PSINDB regularly updated with at least an annual update of each source database to be in line with the progress of the interaction, structural and functional data. We are also dedicated to keep annotating new interactions by our curation team, while we will consider extending the annotation process to new areas, such as mapping binding regions from orthologous proteins, adding new postsynaptic proteins and others.

The field of structural biology greatly changed with the development of AlphaFold2 (42) by Deepmind, and the scientific community already utilized it for more specific tasks (43), including protein complex prediction, disorder region prediction or functional characterization. These applications will likely replace current state-of-the-art methodologies and integrating them into PSINDB will greatly increase the accuracy of presented data.

We also plan to add new functions and panels to PSINDB. The composition of PSs and their PSD units are important for synaptic plasticity maintenance. A promising direction to open prospects to postsynaptic processes is to include pathway information and directed interactions (44).

## Conclusion

While neuronal function is born out of the collective interaction patterns of the constituent proteins, no large-scale PS specific interaction database exists as of yet. PSINDB also includes information about the binding regions, which is not readily available in most PPI databases and is of special importance for the multivalent interactions common in the PS. In addition, this information can be easily compared with structural and functional annotations. This aspect is key for both experimental design for further studies and *in silico* modeling of postsynaptic complexes. PSINDB not only offers an easy-to-use browsable interface but also contains standardized download formats that can be imported into popular applications such as Cytoscape (45).

## Methods

### Selection of postsynaptic proteins

We downloaded the human, mouse and rat reference proteomes (2021_July release) from UniProt (21). We assigned orthologous proteins using the OMA database (46) and gene names. To define postsynaptic proteins, the following sources were utilized: SynaptomeDB (15), Genes2Cognition (16), SynGo (14) and GeneOntology (13) PS/postsynaptic terms (and their child terms). Furthermore, we also display protein expression data from Human Protein Atlas (40); however, we are not using it as a filter. Proteins showing neuronal expression in Cerebral cortex, Caudate or in the Hippocampus are displayed.

### Manual curation

For the first step in the curation, we extensively searched PubMed and Google scholar for relevant publications. Since many of these publications were already included in other PPI databases, we only used articles that were not processed elsewhere. The curation work was done adhering to the HUPO-PSI Molecular Interaction (MI) workgroup standards, and at least MIMIx curation level was used (47), while terms describing interactions were taken from the PSI-MI ontology (https://www.ebi.ac.uk/ols/ontologies/mi). We considered every described interaction between rat, mouse and human proteins and then mapped them back to the human reference proteome.

### Data integration

We integrated binary PPI information from the IntAct (17), BioGrid (18) and STRING (20) databases. Furthermore, we derived interaction data from PDB (19): first we reconstructed the full structure using the BIOMT records, and then Voronota (48) was used to calculate intermolecular surfaces and to identify interacting residues. Although all sections of the database are limited to PS–PS protein interactions, in the ‘all partners’ view, non-PS proteins are displayed as well.

We used the following protein structure-related information: transmembrane segments (Human Transmembrane Proteome database (49)), liquid–liquid phase separation (PhaSePro database) (50), short linear motifs (Eukaryotic Linear Motifs database) (51), phosphorylation (UniProt annotations), domains (PFAM database) (52), coiled-coils (Deepcoil prediction method (53) and UniProt annotations), disordered regions and disordered binding regions (IUPred2A prediction method (54)) and disease-causing germline mutations (from OMIM database (22)) classified in Disease Ontology (55). Protein alignments between isoforms were done using ClustalOmega (56).

### Defining binding regions

For each experiment, we encoded the properties of the interacting region using terms from the HUPO-PSI MI Ontology. In the case of PDB-derived interactions, the constructs were defined as sufficient for binding (in line with the curation policies), and we introduced an additional term for atomic contacts that could not be fitted onto the ontology.

When collecting these regions from different experiments for the same interaction, all binding regions were merged, and the ones with the most specific information were used (i.e. atomic contact > necessary for binding > sufficient for binding > binding-associated regions). Similarly, when binding regions of a protein with different partners were merged, all regions were included, and the most specific one was displayed.

### Fingerprints

We used GeneOntology and DiseaseOntology to define fingerprints: first we mapped back the ancestor terms of each annotation to the corresponding protein and then counted how many times different terms appear in the network of the protein. This procedure was done separately for GO:Biological Process, GO:Molecular Function and Disease Ontology trees.
